# Video Slice: Image Compression and Transmission for Agricultural Systems

**DOI:** 10.3390/s21113698

**Published:** 2021-05-26

**Authors:** Takaaki Kawai

**Affiliations:** Graduate School of Science and Technology, Shizuoka University, Hamamatsu City 432-8011, Japan; kawai.takaaki.19@shizuoka.ac.jp

**Keywords:** agriculture, image file compression, Internet of Things

## Abstract

When agricultural automation systems are required to send cultivation field images to the cloud for field monitoring, pay-as-you-go mobile communication leads to high operation costs. To minimize cost, one can exploit a characteristic of cultivation field images wherein the landscape does not change considerably besides the appearance of the plants. Therefore, this paper presents a method that transmits only the difference data between the past and current images to minimize the amount of transmitted data. This method is easy to implement because the difference data are generated using an existing video encoder. Further, the difference data are generated based on an image at a specific time instead of the images at adjacent times, and thus the subsequent images can be reproduced even if the previous difference data are lost because of unstable mobile communication. A prototype of the proposed method was implemented with a MPEG-4 Visual video encoder. The amount of transmitted and received data on the medium access control layer was decreased to approximately 1/4 of that when using the secure copy protocol. The transmission time for one image was 5.6 s; thus, the proposed method achieved a reasonable processing time and a reduction of transmitted data.

## 1. Introduction

Internet of Things (IoT) and artificial intelligence (AI) have gained considerable research attention in the field of agricultural work automation. For example, methods for counting fruits [1] and flowers [2] using field images have been investigated to predict the yield of crops. Methods used to predict the required amount of irrigation based on cultivation field images have also been studied [3]. Cultivation field images are required to replicate the crop observation task performed by farmers manually.

The more complicated the cultivation environment recognition is when using AI, the higher the computing power required. When the cultivation field is in a greenhouse or in an outdoor environment, it is difficult to install precision equipment. Therefore, integrating AI and cloud computing has become a practical approach. When an AI-based system runs on a cloud, devices on the cultivation field can be used to transmit the image files to the cloud; however, most cultivation fields tend to lack communication infrastructure. Therefore, these devices transmit the image files using a mobile communication network. This leads to a high operating cost owing to the large amount of data that must be transmitted over the pay-as-you-go mobile communication service. As an example, let us assume that an image of one plant is captured once each minute. When the shooting time is 12 h/day and the image size is 1920 × 1080, the amount of image data per month is approximately 30 GB. If we capture pictures of multiple plants, the amount of data will increase in proportion to the number of plants. Therefore, a method that can minimize the amount of transmitted data is required.

Cultivation field images have the characteristic that the landscape does not change considerably besides the appearance of the plants. This characteristic can be exploited to minimize image data. Reducing the data size of images that have only a few landscape changes has been researched in the field of remote desktop systems. There are three approaches to reducing image data. The first is transmitting only meaningful data, as executed by the X Window System [4] and Microsoft’s Remote Desktop Protocol (RDP) [5]. However, in the case of agricultural systems, the data generated by this approach would only be available for a specific purpose (e.g., only for detecting leaf wilting or counting flowers). Therefore, this approach is not suitable for a general-purpose monitoring system. The second approach is to transmit images only when they include a difference [6]. However, it is difficult to identify agricultural images that do not have mechanical changes because the changes in plants are insignificant. The third approach is to use a video encoder to calculate the difference data of two images and then transmit the difference data irrespective of whether the images have differences [7,8]. While these methods can be applied to agricultural monitoring systems, they stack image difference data, making it difficult to restore the subsequent image files transmitted when data are lost by mobile communication disconnection. Thus, these methods should be used in a manner that does not affect the subsequent images after data loss.

In this paper, the author proposes a method to transmit image difference data and support restoring of subsequent image files transmitted after a loss of difference data. The remainder of this paper is organized as follows. Section 2 describes a hypothetical cultivation experiment as a case study. In Section 3, the related work is reviewed. Section 4 presents the proposed method and Section 5 evaluates the proposal. Section 6 comprises detailed discussion on the research outcomes. Finally, Section 7 describes the conclusion.

## 2. Case Study

**Figure 1:**
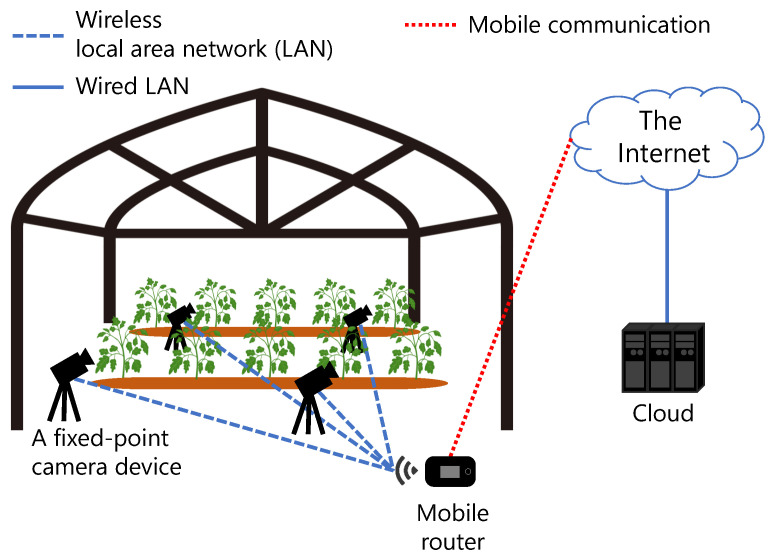
Assumed cultivation experiment. Fixed-point camera devices generate image files of plants in a greenhouse and transmits these files to the cloud.

The hypothetical experiment is an automatic irrigation experiment performed in a greenhouse as shown in Figure 1. A fixed-point camera is used to photograph one plant.

The images are used to observe leaf wilting caused by water shortage; these images need to be of sufficient quality to allow farmers to determine the cultivation scenario. Although the images should not be out of focus, there is no need for high-definition images; details such as the leaf surface are not required. Further, direct sunlight hardly enters the roof because the greenhouse is covered with translucent vinyl; however, the brightness of the landscape changes because of light other than direct sunlight. In addition, farmers often appear in the images several times a day. The images are captured once every minute during the daytime. In most cases, the shooting time is between 6 AM to 6 PM. The camera transmits the images to the cloud using a mobile router connected to a wireless local area network (LAN). The shooting duration is over 1–2 months, which is the general vegetable cultivation period. The number of fixed-point cameras is assumed to be approximately 4–8 per greenhouse.

## 3. Related Work

### 3.1. The Importance of Remote Monitoring System and Transmitted Data Reduction

Cultivation field images play an important role in both technology and business. From a technological aspect, field images are used for the automation of cultivation. For example, they are used for counting fruits [1] and flowers [2], which is important to predict the crop yield. Images of cultivation fields can also be used for irrigation control [3]. From a business perspective, the data from agricultural IoT systems are an important factor for the evaluation of agricultural finance. For example, investors and banks make investment and loan decisions based on historical cultivation data provided by the IoT systems [9].

Image monitoring systems can be developed to work with the cloud by continuously uploading images, or work in a stand-alone mode. If the focus is on challenges such as computational power, technical support, and cyber security, an image uploading system is preferred. Novel cultivation approaches such as those using AI tend to require systems with high computational performance such as a precision apparatus. However, cultivation fields are affected by harsh environmental phenomena such as high solar radiation, extreme temperatures, rain or high humidity, and strong winds [10]. Therefore, the number of on-site equipment in cultivation fields must be reduced to achieve robustness.

The processing of data on the cloud is also effective for improved technical support and cyber security of the developed algorithms and cultivation data management. Furthermore, farmers may not necessarily have sufficient knowledge to operate an IoT system [11,12]. On-site IoT devices could be exposed to the risk of fraudulent access, which may lead to leakages of agricultural secrets [9]. By contrast, a cloud-based system is managed by reliable technical staff to protect the cultivation algorithm and data from theft, cyber attacks, and unauthorized access. Further, cloud-based systems are also convenient for remote technical support.

Agricultural systems that utilize the cloud have been reported. Liu et al. [13] proposed using the cloud for an agricultural remote monitoring system that collects video data. Kim et al. [14] proposed a cloud-based system that focused on strawberry disease prediction using image data. Morais et al. [15] proposed a cloud data management system to support the collection of image data. This data management system assumes that images are collected by a drone; however, this system can be used for field monitoring using fixed-point cameras.

For communication with the cloud over the Internet, wireless LAN, WiMAX, and cellular network can be used [10,16]. In the absence of a network infrastructure, WiMAX and cellular networks are the practical options. Especially, cellular networks can be used when both wireless LAN and WiMAX are not available. Shafi et al. [16] also reported that a cellular network is suitable when high data rate communication is required. However, a cellular network is often a pay-as-you-go service with high operational costs. Therefore, it is important to reduce the amount of data transmitted over the cellular network. Jawad et al. [17] focused on reducing power consumption; however, they also reported the importance of data reduction.

### 3.2. Remote Monitoring System

Remote monitoring systems have been studied in many areas such as unmanned aerial systems (UAS) applications, wildlife monitoring, cultivation monitoring, and video surveillance systems for security.

Research on UAS has focused on stable connection for communication. Specifically, integration with air traffic control (ATC) has been researched as it is important for safety [18]. In the image processing domain, researchers working on UAS have mainly focused on reproducing or analyzing on-ground objects [19]. For example, scanning post-disaster buildings [20] and deformation measurement of a large-scale solar power plant [21] have been studied. Thus, the research on transmitted data reduction has not been the main focus.

Researchers working on wildlife monitoring consider the large amount of image data as a problem. However, they have been addressing this problem by focusing on data management methods, easy-to-use data formats, and cloud storage platforms [22]. In the field of image capturing systems, the main topic of interest has been addressing the problem of imperfect detection wherein animals present within a sampling area are not always detected [23].

In the case of cultivation monitoring, data extraction from images has been the main topic of interest among researchers. For example, object classification and disease detection (e.g., flower counting and disease diagnosis) have been studied [24].

In the field of security, wireless LAN and WiMAX, which are not pay-as-you-go services, are often used for communication [25]. Therefore, monitoring systems for security neglect the amount of data being transmitted. Transmitting large amounts of video data is challenging; however, load balancing with mobile edge computing has been proposed as a practical solution [26] for large scale systems. Therefore, transmitted data reduction has not been the main focus in the research on remote monitoring systems.

### 3.3. Image Data Reduction

Both cultivation field images and personal computer screen images do not change the landscape considerably. Therefore, transmitting the cultivation images is similar to image transmission by remote desktop systems. To minimize the amount of image data, three approaches have been proposed in the field of remote desktop systems.

The first approach is transmitting only meaningful data. For example, X Window System [4] and RDP [5] transmits only image drawing commands instead of the image itself. The program that receives these commands reproduces the screen image. Extracting meaningful data from images can be applied to agricultural systems. For example, optical flow such as Farneback [27] or object detection by a deep neural network (DNN) such as Mask R-CNN [28] has the possibility of generating data that represent leaf wilting. However, this approach generates data that are available only for a specific purpose (e.g., leaf wilting detection or flower counting) and it is difficult to build a general purpose monitoring system; therefore, it must transmit the entire image file.

The second approach is transmitting images only when the images show a difference. Yang et al. [29] reported that Miracast [6]—one of the technologies to transmit screen images—does not transmit data if there is no change in the images. This method can be interpreted as not transmitting images when there is no change in the cultivation field image in the case of agriculture automation. However, it is difficult to identify images that do not have mechanical changes because the changes in plants are minute.

The third approach is transmitting the difference data of two images irrespective of whether the images have any differences. Thai et al. [7] and Zhang et al. [8] used a video encoder to generate the difference data of screen images. Thai et al. proposed a method that compresses images using a video encoder if the amount of image data does not fit the bandwidth of the network. The H.264/AVC standard [30] was used as the video encoder. Zhang et al. achieved a high compression ratio by exploiting the fact that the color of the screen images is not abundant. This method quantizes the image with the HSV color space in advance, and then, it encodes the image using H.254/AVC. These methods can be applied to agricultural monitoring systems. A similar method that transmits the difference data between images generated by an existing video encoder is proposed in this paper.

Zhang et al. [31] cited H.264/AVC, H.265/HEVC [32], and VP9 [33] as typical video encoders. MPEG-4 Visual (MPEG-4 part 2) [34] was also cited as a typical compression method of the previous generation. A preliminary experiment revealed that the MPEG-4 Visual video encoder can be used on devices with limited computing power (e.g., Raspberry Pi Zero W [35]) with a short processing time. These video encoders minimize the total amount of image data by recording the difference data of images created from images captured at adjacent times. Put simply, these methods heap the difference data. In such cases of heaping data, it is difficult to restore the subsequent image files transmitted after a data loss caused by communication disconnection. However, the transmission of these data can be unstable when mobile communication is used in a cultivation field. Thus, these methods should be used in a manner that does not affect the subsequent images after data loss.

In the field of image processing, several studies have investigated a method to minimize the amount of data in a single image. Toderici et al. [36] proposed a method that minimizes the amount of data using a DNN-based encoder and decoder. This method extracts the features of an image using the encoder, and the decoder restores the image by generating data minimized by the encoder. Krishnaraj et al. [37] achieved a high compression ratio by combining DNN and the discrete wavelet transform. Deep learning tends to require high computational power, however, a method using a convolutional neural network proposed by Prakash et al. [38] was implemented in a microcomputer by Gia et al. [39]. Other image compression methods have also been proposed for hardware with low computational power. For example, Lee et al. [40] realized an image compression method with a low memory consumption for IoT devices. Azar et al. [41] realized an image compression method that can be used on devices for a wireless body sensor network. These methods can achieve effective image compression. However, implementing these methods from scratch is not realistic in terms of learning costs. Therefore, it is reasonable to use standardized image compression methods until these novel methods are provided as libraries.

Table 1 summarizes the aforementioned transmitted data reduction approaches. This study aims to accomplish the adaptivity for agriculture, general versatility, robustness to loss of data, and ease of implementation.

## 4. Proposed Method

### 4.1. Basic Concept

A method that transmits an image file as reference only once at the beginning of the day and then transmits the difference data based on the first image is proposed in this paper. The image data for reference are called the base image, and this data file is called the base image file. The image data that are the transmission target besides the base image are called the target image, and this data file is called the target image file. The base image is the first image photographed every day. The difference data are obtained using the base image file and the target image file. In the case of heaping the difference data generated from the images captured at adjacent times, such as in conventional video encoders, it is difficult to restore the subsequent images if the difference data are lost due to communication disconnection. When the image captured at a specific time is used as the reference image, the subsequent image can be restored even if the difference data are lost. Thus, the loss of difference data does not affect the subsequent images. This characteristic allows minimizing the image file loss even when using mobile communication, which tends to be unstable.

**Figure 2:**
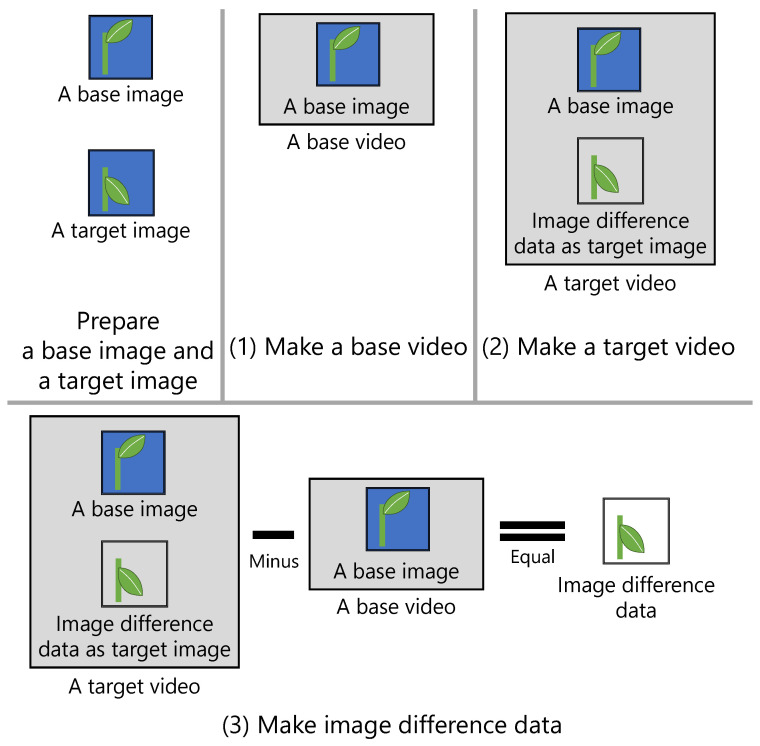
Steps for generating the difference data between images. The difference in the binary data between a target video and a base video is treated as the difference data between the images.

Figure 2 shows the steps for generating the difference data of images.

An existing video encoder is applied to generate the difference data. First, a video file containing only a base image is generated. This video data are called the base video, and its file is called the base video file. Then, a video file containing the base image and a target image is generated. This video data are called the target video, and its file is called the target video file. Finally, the difference data between the images are generated by calculating the difference in the binary data between the base video file and the target video file.

The differences that can be recognized by the human eye are considered as the difference data. However, this criterion is qualitative, and it is necessary to detect the difference using a quantitative approach. Since the quantification of differences that are recognizable to the human eye has been studied in the field of video encoders, the author applied the existing criteria by using a video encoder in this study.

**Figure 3:**
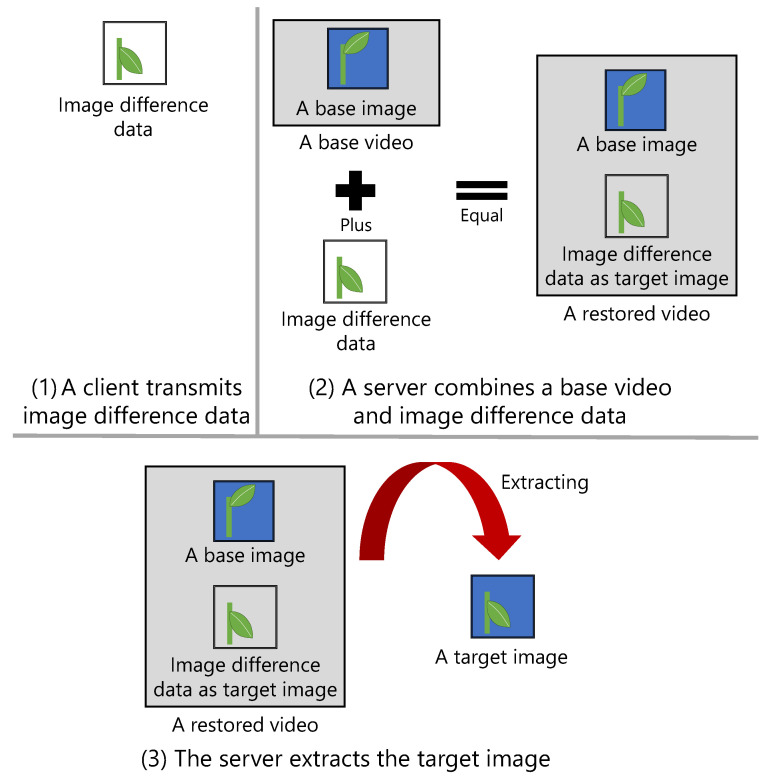
Steps for transmitting an image between a client and a server. The target image is extracted from a restored video, which comprises an in advance received base video and the difference data between the images.

The transmitter of images is the client, and the receiver is the server. Figure 3 shows the steps involved in image transmission between the client and the server.

First, the client creates a target video file each time an image is captured, and it transmits the difference data between the base video file and target video file to the server. If this transmission is the first one for the day, the base video file is also transmitted to the server. Then, the server combines the received difference data with the base video file. These video data are called restored video, and its file is called a restored video file. Finally, the server extracts the last image of the restored video file and saves it as a target image in the Joint Photographic Experts Group (JPEG) file format. This image transmission method is called a “video slice”.

### 4.2. Prototype

**Figure 4:**
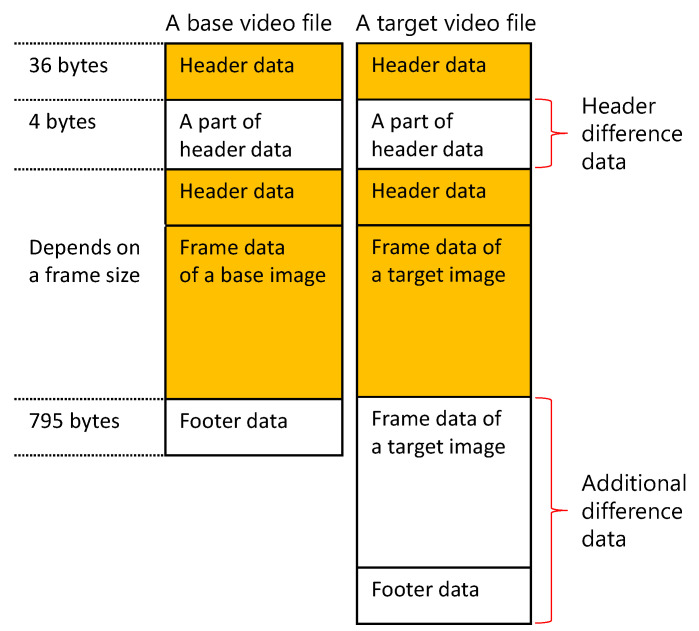
Characteristics of video file binary data. When an image is added to a video, a difference is generated between the part from the header data and the part from the footer data of the base video.

A prototype of the video slice was implemented by employing an MPEG-4 Visual video encoder. Video slice can be realized using other video encoders; however, the MPEG-4 Visual video encoder was selected because it can be used even on devices with low computational power, as described in Section 3. The binary data of the base and target video files show the characteristics illustrated in Figure 4 when MPEG-4 Visual is used.

For the sake of convenience, we assume that a video file comprises header data, frame data, and footer data. A frame indicates the image data in a video file. The colored part of Figure 4 represents the same data included in the base video file and the target video file.

The data difference appears at two instances. The first is 4-byte data in the 36th byte from the beginning. The second is the data starting at the 795th byte from the end of the base video file. The first difference data point is called the header difference data, and the second one is called the additional difference data. The video slice prototype uses these two data points as the set of difference data. The set of difference data is referred to as the image difference data in this paper.

**Figure 5:**
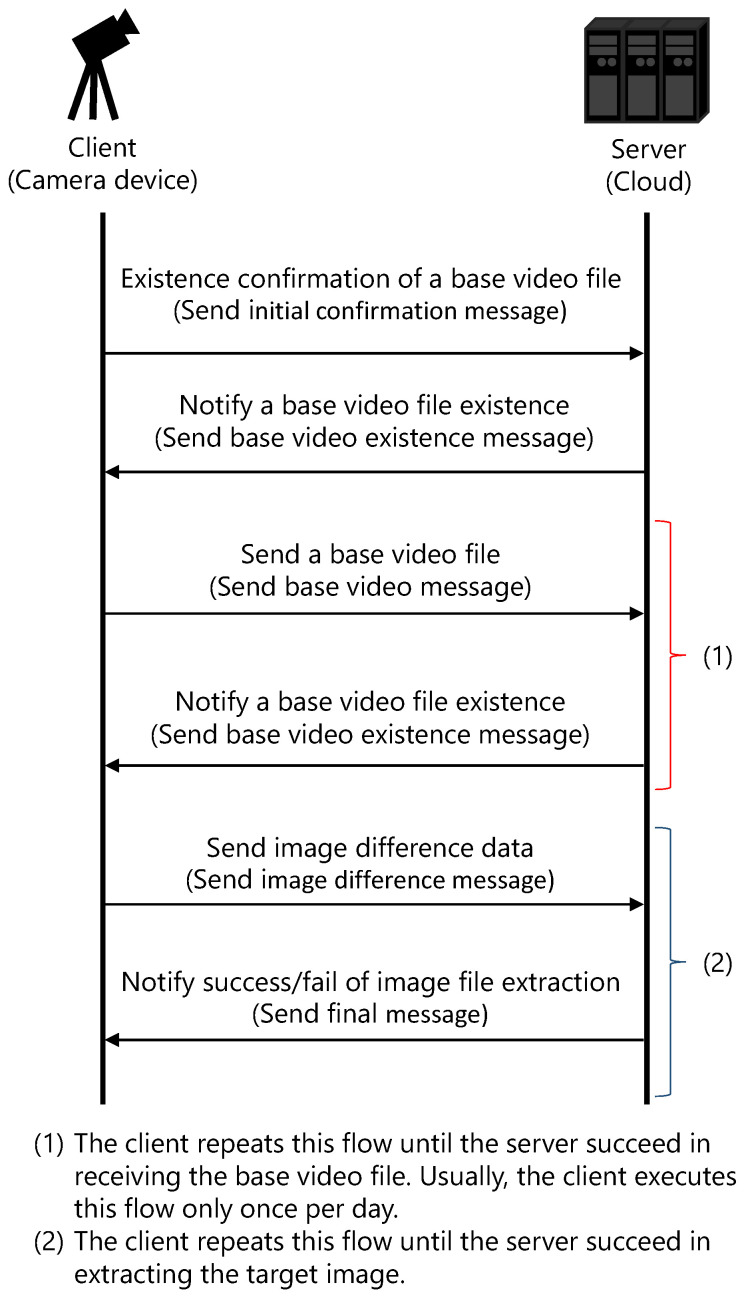
Communication flow. The base video file and the image difference data are transmitted the first time, and only the image difference data are transmitted the second time.

The communication flow between the client and the server is shown in Figure 5.

The camera device is assumed to be the client, and the cloud is the server. First, the client sends a request to the server to check for the existence of the base video file by transmitting an initial confirmation message. The server then notifies the client about the existence or nonexistence of the base video file by transmitting a base video existence message. If the server does not have a base video file, the client transmits the base video file with a base video message. The base video file is created and transmitted only once at the time of sunrise in the usual case. The server transmits the base video existence message again. When the server notifies the client about the failure of base video reception, the client re-executes the flow from the transmission of the base video message. Next, the client transmits the image difference data to the server using an image difference message. The server combines the base video file and the image difference data, and then, it extracts the target image. The extracted target image data are saved as a JPEG file. Finally, the server notifies the client regarding the status (success or failure) of target image extraction by transmitting the final message. If the server notifies the failure of target image extraction, the client re-executes the flow from the transmission of the image difference message.

The ease of implementation was given considerable importance during the development of the prototype of video slice. Therefore, this communication is performed on the transmission control protocol/internet protocol (TCP/IP), and the retransmission is executed by TCP/IP when a temporary transmission error occurs. One server program does not accept simultaneous connections by multiple clients. The transmission of the base image itself is realized by creating a target video file that contains two base images. This transmission technique unifies the transmission process of the base and target image files.

**Figure 6:**
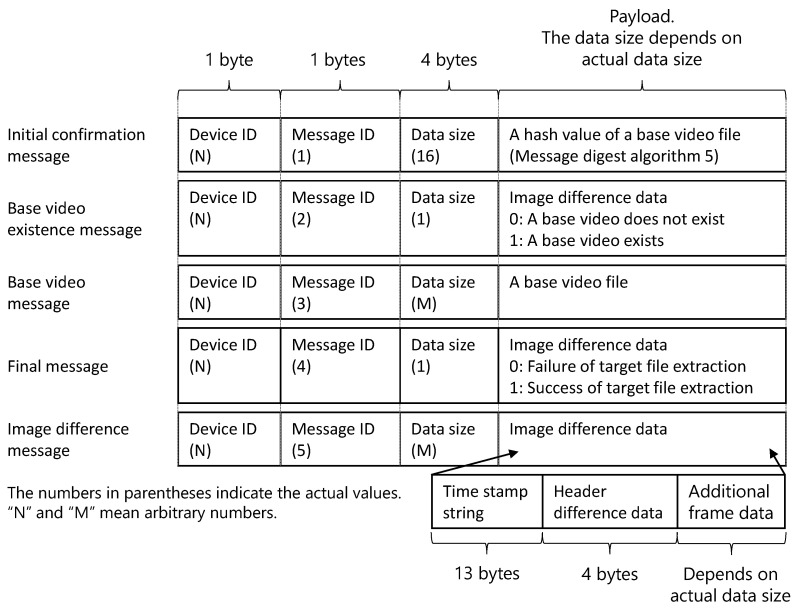
Data format of the messages. It comprises device ID, message ID, data size, and payload, which are the main data.

Figure 6 shows the data format for each message.

Each message comprises a device ID, message ID, data size, and payload, which are the main data. The device ID is used to identify the client. However, the server of the prototype does not support multiple clients identified by device IDs because the implementation is simplified. The message ID is used to identify the message. The numbers in parentheses indicate the actual values. Message IDs 1–5 are assigned in ascending order. The data size indicates the size of the payload. To receive an entire message via TCP, the device ID, message ID, and data size are received first, and then, a payload is received based on the data size.

The payload depends on the type of message. In the case of the initial confirmation message, the hash value of a base video file is stored in the payload. The message digest algorithm 5 is used as the hash algorithm. The server confirms the base video existence by comparing the hash value of the existing video file and the received hash value. For the base video existence message, the value of 0 or 1 is stored in the payload, wherein 0 indicates that the server does not have the base video file, and 1 indicates that the server has the base video file. For the base video message, the binary data of the base video file are stored in the payload. For the final message, the value of 0 or 1 is stored in the payload. Here, 0 represents failed target image extraction, and 1 represents successful target image extraction. For the image difference data message, in addition to the aforementioned header difference data and additional difference data, a character string representing the timestamp of the image recording time is also stored in the payload. A timestamp is used for the file name assigned to the JPEG file when the target image is restored. The timestamp format is “YYYYmmdd_HHMM” where “YYYY” shows the year, “mm” shows the month, “dd” shows the day, “HH” shows hour, and “MM” shows minute.

A Raspberry Pi Zero W (The Raspberry Pi Foundation, Cambridge, UK) simulates the camera device, and a Raspberry Pi 4 Model B (The Raspberry Pi Foundation, Cambridge, UK) [42] simulates the cloud. The Raspberry Pi series is a commonly available microcomputer series. The Raspberry Pi Zero W has a relatively low computing power among the Raspberry Pi series and is used for imitating IoT devices. The Raspberry Pi 4 Model B has relatively high computing power among the Raspberry Pi series and is used for imitating a cloud. The prototype of video slice is implemented in Python 3 [43], which is an easy-to-learn programming language. The MPEG-4 Visual encoder implemented in the image processing library called OpenCV [44] is used as the video encoder. The total number of lines of source code was approximately 450 for the author’s prototype (including client and server programs), which is easily manageable for an individual programmer. Table 2 summarizes the specifications of the hardware used to implement the prototype of video slice.

## 5. Evaluation

### 5.1. Condition of Communication and Image Files

The communication between the Raspberry Pi Zero W and the Raspberry Pi 4 Model B was performed inside the LAN (which was close to the ideal conditions) to unify the conditions for each communication trial as far as possible. The Raspberry Pi Zero W was connected to the router (Archer C6, TP-Link Corporation Limited, Shenzhen, China) via wireless LAN (IEEE 802.11n, 72.2 MB/s bandwidth), and the Raspberry Pi 4 Model B was connected to the router via wired LAN. The images to be transmitted were obtained in advance in an environment that simulated the cultivation field shown in Figure 1. Bean sprouts were used as subjects, which grow relatively quickly. The growth process of bean sprouts was photographed over one week. The bean sprouts were placed near a window with a thin curtain, and the environment was simulated wherein sunlight besides direct sunlight gradually changes the brightness in the image. The images were JPEG files of size 1920 × 1080 and quality 100. The extracted target image was also saved as a JPEG file with quality 100.

### 5.2. Reduced Data Amount

The amount of transmitted and received data was measured on the server after image files (720 images) for one day were transmitted via the secure copy protocol (SCP), video slice, or TCP with H.264/AVC. The received data amount and the transmitted data amount are measured on the server because both communication directions are subject to billing in mobile communication. SCP is a widely used protocol for transferring files to remote computers. SCP uses the TCP, similar to the video slice prototype. H.264/AVC is used by Thai et al. [7] and Zhang et al. [8]. Thai et al. recorded videos at 25 frames per second (fps) and Zhang et al. recorded videos at 30 fps. To reproduce these methods, the author made a video file at 25 fps from the image files using H.264/AVC and this video file was transmitted by TCP. The amount of transmitted and received data was measured by running the packet capture application Wireshark [45] on the Raspberry Pi 4 Model B. Images were transmitted from the Raspberry Pi Zero W to the Raspberry Pi 4 Model B, and Wireshark recorded the total transmitted and received data amount in the medium access control (MAC) layer.

**Figure 7:**
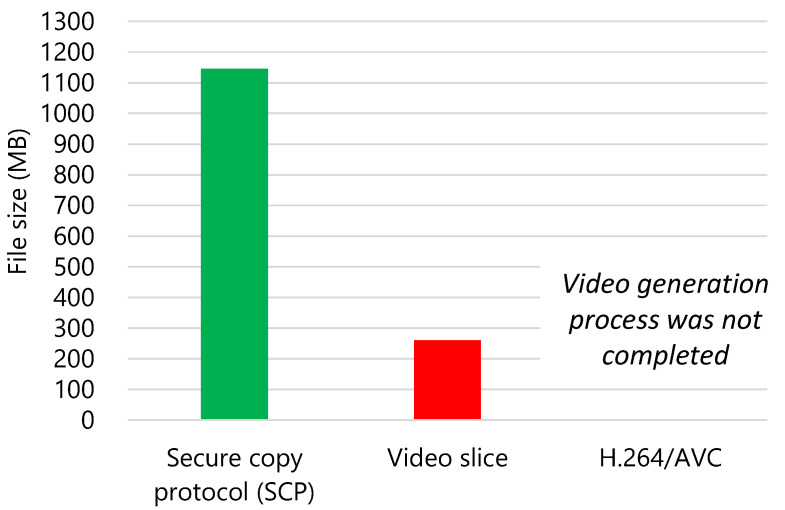
Total transmitted and received data amount in the medium access control layer. The data amount of the video slice was approximately 1/4 of that of SCP.

Figure 7 shows the result of the data amount measurement.

The video generation process of H.264/AVC was not completed because the central processing unit (CPU) lacked the required computational capability. The methods of Thai et al. and Zhang et al. could not be compared on the hardware that simulates the low computational power of an agricultural IoT device.

The data amount of SCP was 1145 MB and that of the video slice was 261.1 MB. The data amount of video slices became approximately 1/4 of that of SCP. Therefore, video slice achieves efficient image compression. Further, the data reduction amount of the image data itself was also measured. The size of base video file and the data amount of the additional difference data in the image difference message were measured. The data amount of the original image files was 1088 MB and that of the video slice was 250.1 MB. The amount of image data was reduced to approximately 1/4 by using video slice. Therefore, image compression was achieved.

### 5.3. Transmission Processing Time

The processing time of one target image transmission was measured in the case of the SCP and the video slice. The image compression by H.264/AVC that simulates Thai et al. [7] and Zhang et al. [8] was not included in this evaluation because H.264/AVC did not work on the Raspberry Pi Zero W as described on Section 5.2. The time from the beginning to the end of the SCP command was defined as one transmission processing time in the case of SCP. The time from the beginning to the end of the client program was defined as one transmission processing time for video slice. The processing times of the initial and usual transmissions were measured for video slice. The initial transmission includes transmitting the base video file and the image difference data. The usual transmission includes the transmission of only the image difference data.

The measurement result was obtained using an average of 10 measurements. Different images were transmitted in 10 trials to reduce the bias in the transmission time. However, the same 10 types of images were transmitted by SCP and video slice. In particular, SCP transmitted the first to tenth images to the server sequentially. For the initial transmission using video slice, one image was treated as both the base image and the target image, and the base video file and the image difference data were transmitted. This transmission was repeated for all 10 images. For the usual transmission of video slice, the first image was used as the base image, and the first to tenth images were used as the target images sequentially.

**Figure 8:**
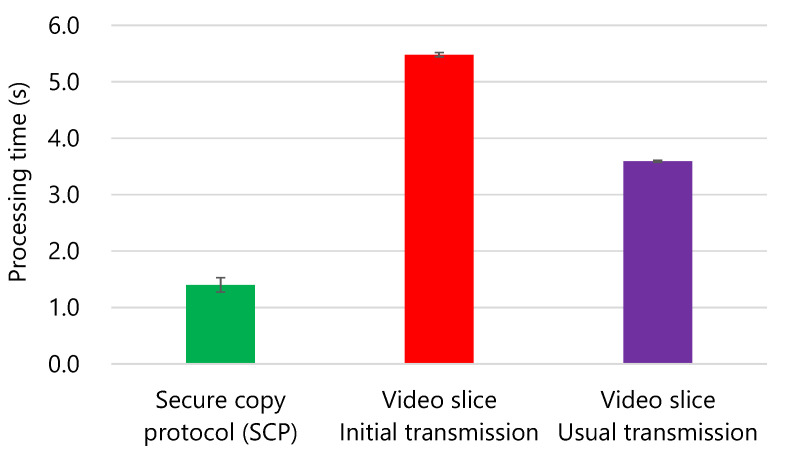
Processing time per target image. The execution time of the video slice was 5.6 s at the maximum, and thus, the video slice could transmit an image once per minute.

Figure 8 shows the results of the transmission processing time.

The error bar represents the standard error. The SCP took 1.4 s, the initial transmission of video slice took 5.6 s, and the usual transmission of the video slice took 3.6 s. The transmission processing time of the video slice was approximately 3.1 times that of SCP in the case of the initial transmission, which requires the longest time. However, the processing time of video slice was sufficiently practical because it was assumed that one camera node transmits only one image in one minute.

In this prototype, the base video and image difference data created from the same image file are transmitted when the initial transmission is executed to simplify the implementation. Two video file generation processes of the base video file and target video file are executed. Therefore, the transmission processing time during the initial transmission was approximately 1.5 times that of the usual transmission. If this prototype were improved to transmit only the base video file and extract the base image from that base video file, the processing time would be decreased to be the same as that of the usual transmission.

The standard error was 0.7% of the average for the initial transmission, which showed the maximum standard error. Therefore, the processing time for each trial was almost the same.

### 5.4. Image Degradation

Image degradation after the transmission of one day’s image file was measured. The peak signal-to-noise ratio (PSNR) and structural similarity (SSIM) are typical indexes for evaluating the degree of image degradation. Further, the result of PSNR and SSIM are shown in this paper. Kobako [46] described a guideline for PSNR and SSIM. Table 3 lists this guideline. The results of PSNR and SSIM are summarized in Table 4.

The amount of data is minimized by deleting pixel information that cannot be recognized by the human eye when using the MPEG-4 Visual video encoder. Therefore, degradation occurs when an image is transmitted using video slice. However, the minimum PSNR was greater than 30 dB, and the minimum SSIM was also greater than 0.90. Therefore, the degradation of the images was invisible to the human eye unless the images were enlarged.

**Figure 9:**
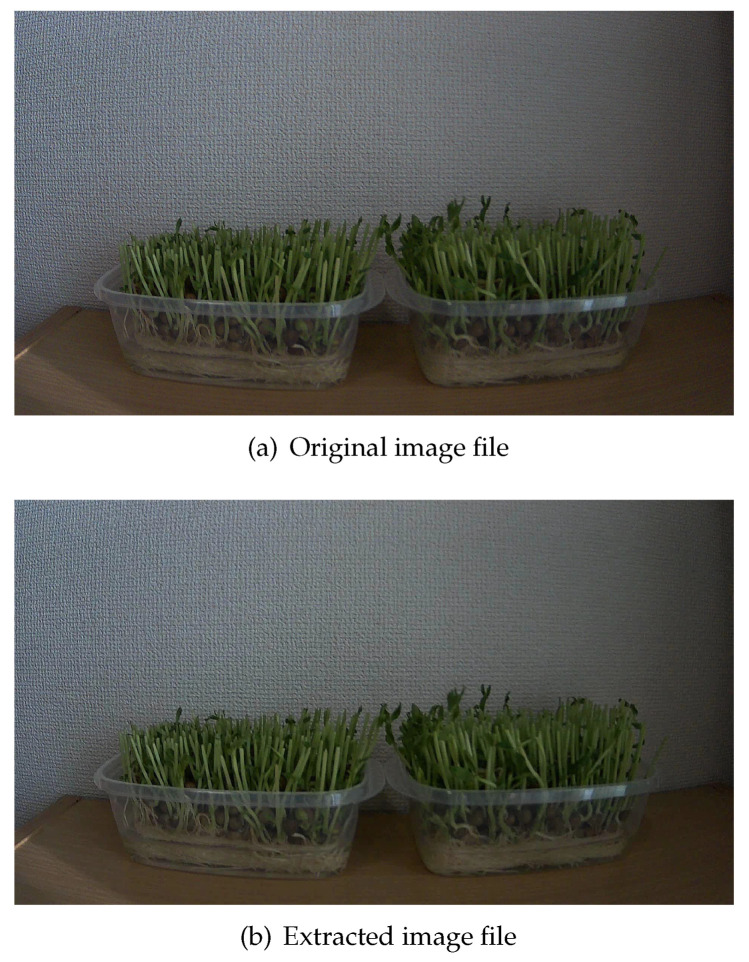
Image before compression and that extracted from the video file. Even if the SSIM is the lowest, the degradation of the image is invisible to the human eye.

The image degradation was confirmed via visual inspection as well. The image with 0.93 SSIM, which is the minimum SSIM, is shown in Figure 9.

Figure 9a is the original image, and Figure 9b is the image extracted from the base video file and image difference data.

The image was dark because the weather at that time was such that the sunlight was blocked out. A visual comparison suggested that there was no difference between the two images when the SSIM was 0.93. Thus, the video slice achieves an image quality that meets the requirements for monitoring the cultivation field.

**Figure 10:**
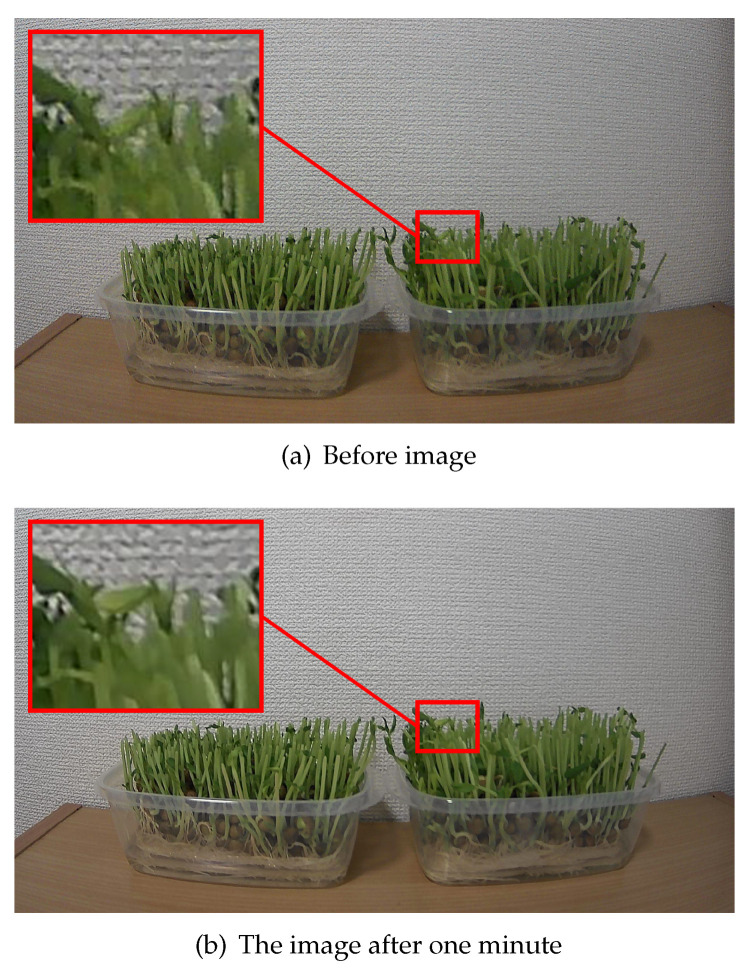
Images containing human-perceivable differences. The leaf in the area enclosed by the square is enhanced in size for improved focus.

Figure 10 shows the images containing human-recognizable differences for reference. It can be noticed that the leaf in the area enclosed by the square (enlarged for understandability) was growing.

### 5.5. Worst Case Evaluation

**Figure 11:**
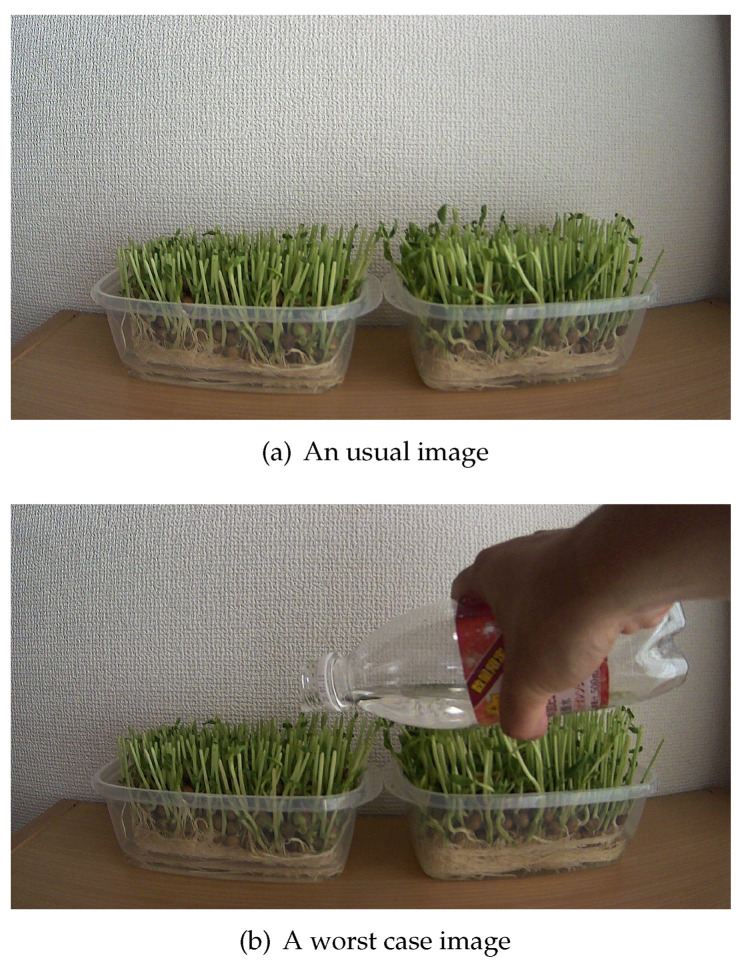
A usual case and worst case image. To simulate the situation that a farmer was captured every two minutes, the author transmitted the worst case image once in two minutes.

The situation in that a farmer was captured every two minutes was assumed as the worst case. Figure 11 shows the transmitted images. Figure 11a was one of the usual case images. Figure 11b was the image that captured a farmer’s hand and is referred to as the worst case image.

The author overwrote the every-two-minutes image of the original one day’s images by Figure 11b. 720 images ware transmitted as one day’s image. The amount of transmitted and received data on MAC layer, transmission processing time per one image, and image degradation were evaluated. As a comparative criterion, transmitted and received data amount by SCP was measured.

Table 5 shows the evaluation result of transmitted and received data amount, transmission processing time, and image degradation.

The data amount of SCP was 1156.7 MB and that of the video slice was 217.9 MB. The data amount of the video slices became approximately 1/5 of that of SCP. This compression rate was higher than that described in Section 5.2. The original image file of the worst case was smaller than that of usual case. It was assumed that the hand surface was simpler than the front edge of leaves and the wallpaper of background. These simplification would make the one image compression rate higher. The additional difference data (described in Section 4.2) of the worst image were smaller than the usual image (64% of usual case average). Therefore, the obstacle would not affect the compression efficiency when the surface of the obstacle is simple.

The average transmission processing time of each image was 4.1 and that of the worse case image was 4.0. Therefore, transmission processing time was not increased to a large degree compared to the result described in Section 5.3. The worst PSNR was 30 dB, and the worst SSIM was 0.90. Therefore, the degradation of the images was of a comparable level, as described in Section 5.4. These results show that video slice worked effectively in the worst case.

## 6. Discussion

The proposed method has two restrictions. The first is assuming that when images with slight changes in landscape are transmitted, the amount of difference image data will become smaller. Second, it uses lossy compression, which makes it unsuitable for high-quality image transmission. However, it can be said that this method is appropriate because the landscape of the cultivation field does not change much, and people do not require high-resolution images to confirm the status.

In this study, the author assumed using fixed-point cameras; however, it would be inconvenient to capture a fast-growing plant if the viewing angle is fixed. For such a scenario, a more flexible camera in which the viewing angle can be adjusted by a remote control can be used. In the proposed method, it was assumed that the landscape does not change significantly. However, the prototype system can be improved to make it possible to recapture the base image after changing the viewing angle while maintaining the efficiency of data reduction. Therefore, changing the viewing angle a few times in a day can be made possible in practical operations.

## 7. Conclusions

The author proposed a method to reduce the amount of image data by transmitting only the difference data between the previous image and the current image to the cloud. The learning cost for implementation was low because the image difference data were generated by applying an existing video encoder. Meanwhile, the proposed method is different from the case where only the existing video encoder is used. The image difference data were generated based on the image at a specific time instead of the images with adjacent shooting times. Therefore, the loss of difference data does not affect the restoration of subsequent images. The prototype that used MPEG-4 as the video encoder showed that the transmitting/receiving data amount decreased to approximately 1/4 of that of SCP. Furthermore, the average processing time of transmitting was 5.6 s, which is enough for the transmission of one image per minute.

The proposed method has not been evaluated in an actual greenhouse, which could be the subject of a future study. In the future, the author plans to evaluate IoT-specific advantages such as battery operation, energy autonomy, remote and unattended deployment, and communication services availability.

## Figures and Tables

**Table 1:** Summary of transmission data reduction approaches.

Requirement	Transmitting Only Meaningful Data	Transmitting Only When A Significant Change Appears	Transmitting Difference Data	Compressing One Image	Proposal
Adaptivity for agriculture	Yes	No	Yes	Yes	Yes
General versatility	No	Yes	Yes	Yes	Yes
Robustness to loss of data	Yes	Yes	No	Yes	Yes
Ease of implementation	Yes	Yes	Yes	No	Yes

**Table 2:** Specifications of Raspberry Pi.

Item	Raspberry Pi Zero W	Raspberry Pi 4 Model B
Graphics processing unit (GPU)	Videocore IV 250 MHz	Videocore IV 500 MHz
Central processing unit (CPU)	ARM1176JZF-S (ARMv6) 1 GHz 32 bit single core	ARM Cortex-A72 (ARMv8) 1.5 GHz 64 bit quad core
Random access memory (RAM)	1 GB	8 GB
Storage (Micro SD card)	128 GB	128 GB
Operating system	Raspberry Pi OS 32 bit Release: 2 December 2020 Kernel version: 5.4 No graphical user interface (GUI)	Raspberry Pi OS 32 bit Release: 2 December 2020 Kernel version: 5.4 With GUI
Python3 version	3.7.3	3.7.3
OpenCV version	4.4.0	4.4.0

**Table 3:** Guideline of peak signal-to-noise ratio and structural similarity.

Peak Signal-to-Noise Ratio (PSNR)	Structural Similarity (SSIM)	Subjective Appraisal
Over 40 dB	Over 0.98	Indistinguishable between original and compressed image.
Over 30–40 dB	Over 0.90–0.98	People can notice the degradation when the image is enlarged.
Equal to or lower than 30 dB	Equal to or lower than 0.90	Obviously degraded.

**Table 4:** Degradation of images in a day.

Evaluation Index	Average	Minimum
PSNR (dB)	36.11	34.20
SSIM	0.97	0.93

**Table 5:** Result of the worst case.

Evaluation Index	Result
Transmitted and received data amount of secure copy protocol	1156.7 MB
Transmitted and received data amount of video slice	217.9 MB
Processing time for one image transmission (average of all image)	4.1 s
Processing time for one image transmission (average of worst case image)	4.0 s
Worst PSNR	34.20 dB
Worst SSIM	0.93

## References

[1] Koirala A., Walsh K.B., Wang Z., McCarthy C. (2019). Deep learning—Method overview and review of use for fruit detection and yield estimation. Comput. Electron. Agric..

[2] Chen Y., Lee W.S., Gan H., Peres N., Fraisse C., Zhang Y., He Y. (2019). Strawberry Yield Prediction Based on a Deep Neural Network Using High-Resolution Aerial Orthoimages. Remote Sens..

[3] Barkunan S., Bhanumathi V., Sethuram J. (2019). Smart sensor for automatic drip irrigation system for paddy cultivation. Comput. Electr. Eng..

[4] Scheifler R.W., Gettys J. (1986). The X window system. ACM Trans. Graph..

[5] Microsoft Corporation Remote Desktop Protocol. https://docs.microsoft.com/ja-jp/windows/win32/termserv/remote-desktop-protocol?redirectedfrom=MSDN.

[6] Wi-Fi Alliance Remote Desktop Protocol. https://www.wi-fi.org/downloads-registered-guest/wp_Miracast_Consumer_201301.pdf/7640.

[7] Thai N.Q., Layek M.A., Huh E.N. A Hybrid Remote Display Scheme for Interactive Applications in Band-Limited Environment. Proceedings of the 2017 Seventh International Conference on Innovative Computing Technology.

[8] Zhang A., Wang Z., Han Z., Fu Y., He Z. H.264 Based Screen Content Coding with HSV Quantization. Proceedings of the 2017 10th International Congress on Image and Signal Processing, BioMedical Engineering and Informatics.

[9] Ruan J., Jiang H., Zhu C., Hu X., Shi Y., Liu T., Rao W., Chan F.T.S. (2019). Agriculture IoT: Emerging Trends, Cooperation Networks, and Outlook. IEEE Wirel. Commun..

[10] Tzounis A., Katsoulas N., Bartzanas T., Kittas C. (2017). Internet of Things in agriculture, recent advances and future challenges. Biosyst. Eng..

[11] Elijah O., Rahman T.A., Orikumhi I., Leow C.Y., Hindia M.N. (2018). An Overview of Internet of Things (IoT) and Data Analytics in Agriculture: Benefits and Challenges. IEEE Internet Things J..

[12] Farooq M.S., Riaz S., Abid A., Umer T., Zikria Y.B. (2020). Role of IoT technology in agriculture: A systematic literature review. Electronics.

[13] Liu S., Guo L., Webb H., Ya X., Chang X. (2019). Internet of Things Monitoring System of Modern Eco-Agriculture Based on Cloud Computing. IEEE Access.

[14] Kim S., Lee M., Shin C. (2018). IoT-Based Strawberry Disease Prediction System for Smart Farming. Sensors.

[15] Morais R., Silva N., Mendes J., Adão T., Pádua L., López-Riquelme J.A., Pavón-Pulido N., Sousa J.J., Peres E. (2019). mySense: A comprehensive data management environment to improve precision agriculture practices. Comput. Electron. Agric..

[16] Shafi U., Mumtaz R., García-Nieto J., Hassan S.A., Zaidi S.A.R., Iqbal N. (2019). Precision Agriculture Techniques and Practices: From Considerations to Applications. Sensors.

[17] Jawad H.M., Nordin R., Gharghan S.K., Jawad A.M., Ismail M. (2017). Energy-Efficient Wireless Sensor Networks for Precision Agriculture: A Review. Sensors.

[18] Colomina I., Molina P. (2014). Unmanned aerial systems for photogrammetry and remote sensing: A review. ISPRS J. Photogramm. Remote Sens..

[19] Ham Y., Han K.K., Lin J.J., Golparvar-Fard M. (2016). Visual monitoring of civil infrastructure systems via camera-equipped Unmanned Aerial Vehicles (UAVs): A review of related works. Vis. Eng..

[20] Michael N., Shen S., Mohta K., Mulgaonkar Y., Kumar V., Nagatani K., Okada Y., Kiribayashi S., Otake K., Yoshida K. (2012). Collaborative Mapping of an Earthquake-DamagedBuilding via Ground and Aerial Robots. J. Field Robot..

[21] Matsuoka R., Nagusa I., Yasuhara H., Mori M., Katayama T., Yachi N., Hasui A., Katakuse M., Atagi T. (2012). Measurement of Large-Scale Solar Power Plant by Using Images Acquired by Non-Metric Digital Camera on Board UAV. Int. Arch. Photogramm. Remote Sens. Spat. Inf. Sci..

[22] Steenweg R., Hebblewhite M., Kays R., Ahumada J., Fisher J.T., Burton C., Townsend S.E., Carbone C., Rowcliffe J.M., Whittington J. (2017). Scaling up camera traps: Monitoring the planet’s biodiversity with networks of remote sensors. Front. Ecol. Environ..

[23] Burton A.C., Neilson E., Moreira D., Ladle A., Steenweg R., Fisher J.T., Bayne E., Boutin S. (2015). REVIEW: Wildlife camera trapping: A review and recommendations for linking surveys to ecological processes. J. Appl. Ecol..

[24] Weiss M., Jacob F., Duveiller G. (2020). Remote sensing for agricultural applications: A meta-review. Remote Sens. Environ..

[25] Elharrouss O., Almaadeed N., Al-Maadeed S. (2021). A review of video surveillance systems. J. Vis. Commun. Image Represent..

[26] Alsmirat M.A., Obaidat I., Jararweh Y., Al-Saleh M. (2017). A security framework for cloud-based video surveillance system. Multimed. Tools Appl..

[27] Farnebäck G. Two-Frame Motion Estimation Based on Polynomial Expansion. Proceedings of the Scandinavian Conference on Image Analysis.

[28] He K., Gkioxari G., Dollár P., Girshick R. Mask R-CNN. Proceedings of the IEEE International Conference on Computer Vision.

[29] Yang S., Li B., Song Y., Xu J., Lu Y. (2018). A Hardware-Accelerated System for High Resolution Real-Time Screen Sharing. IEEE Trans. Circuits Syst. Video Technol..

[30] Wiegand T., Sullivan G.J., Bjontegaard G., Luthra A. (2003). Overview of the H.264/AVC Video Coding Standard. IEEE Trans. Circuits Syst. Video Technol..

[31] Zhang T., Mao S. (2019). An Overview of Emerging Video Coding Standards. GetMobile Mob. Comput. Commun..

[32] Sullivan G.J., Ohm J.R., Han W.J., Wiegand T. (2012). Overview of the High Efficiency Video Coding (HEVC) Standard. IEEE Trans. Circuits Syst. Video Technol..

[33] Mukherjee D., Bankoski J., Grange A., Han J., Koleszar J., Wilkins P., Xu Y., Bultje R. The latest open-source video codec VP9—An overview and preliminary results. Proceedings of the 2013 Picture Coding Symposium.

[34] International Organization for Standardization (2001). Coding of Audio-Visual Objects—Part 2: Visual.

[35] The Raspberry Pi Foundation Raspberry Pi Zero W. https://www.raspberrypi.org/products/raspberry-pi-zero-w/.

[36] Toderici G., Vincent D., Johnston N., Jin H., Minnen D., Shor J., Covell M. Full Resolution Image Compression with Recurrent Neural Networks. Proceedings of the IEEE Conference on Computer Vision and Pattern Recognition.

[37] Krishnaraj N., Elhoseny M., Thenmozhi M., Selim M.M., Shankar K. (2020). Deep learning model for real-time image compression in Internet of Underwater Things (IoUT). J. Real Time Image Process..

[38] Prakash A., Moran N., Garber S., DiLillo A., Storer J. Semantic Perceptual Image Compression using Deep Convolution Networks. Proceedings of the 2017 Data Compression Conference.

[39] Gia T.N., Qingqing L., Queralta J.P., Zou Z., Tenhunen H., Westerlund T. Edge AI in Smart Farming IoT: CNNs at the Edge and Fog Computing with LoRa. Proceedings of the IEEE AFRICON.

[40] Lee S.W., Kim H.Y. (2018). An energy-efficient low-memory image compression system for multimedia IoT products. EURASIP J. Image Video Process..

[41] Azar J., Makhoul A., Barhamgi M., Couturier R. (2019). An energy efficient IoT data compression approach for edge machine learning. Future Gener. Comput. Syst..

[42] The Raspberry Pi Foundation Raspberry Pi 4 Model B. https://www.raspberrypi.org/products/raspberry-pi-4-model-b/.

[43] Python Software Foundation Python. https://www.python.org/.

[44] OpenCV Team OpenCV. https://opencv.org/.

[45] The Wireshark Foundation Wireshark. https://www.wireshark.org/.

[46] Kobako M. (2011). Image compression guidelines for digitized documents. J. Image Inf. Manag..

